# Rapid High-Resolution Mosaic Acquisition for Photoacoustic Remote Sensing

**DOI:** 10.3390/s20041027

**Published:** 2020-02-14

**Authors:** Saad Abbasi, Kevan Bell, Parsin Haji Reza

**Affiliations:** 1 PhotoMedicine Labs, Department of Systems Design Engineering, University of Waterloo, Waterloo, ON N2L 3G1, Canada; srabbasi@uwaterloo.ca (S.A.); kevan.bell@uwaterloo.ca (K.B.); 2 IllumiSonics Inc., Department of Systems Design Engineering, University of Waterloo, Waterloo, ON N2L 3G1, Canada

**Keywords:** photoacoustic, PARS, mosaic

## Abstract

Mechanical stages are routinely used to scan large expanses of biological specimens in photoacoustic imaging. This is primarily due to the limited field of view (FOV) provided by optical scanning. However, stage scanning becomes impractical at higher scanning speeds, or potentially unfeasible with heavier samples. Also, the slow scan-rate of the stages makes high resolution scanning a time-consuming process. Some clinical applications such as microsurgery require submicron resolution in a reflection-mode configuration necessitating a method that can acquire large field of views with a small raster scanning step size. In this study, we describe a method that combines mechanical stages with optical scanning for the rapid acquisition of high-resolution large FOVs. Optical scanning is used to acquire small frames in a two-dimensional grid formed by the mechanical stages. These frames are captured with specific overlap for effective image registration. Using a step size of 200 nm, we demonstrate mosaics of carbon fiber networks with FOVs of 0.8 × 0.8 mm^2^ captured in under 70 s with 1.2 µm image resolution. Larger mosaics yielding an imaging area of 3 × 3 mm^2^ are also shown. The method is validated by imaging a 1 × 1 mm^2^ section of unstained histopathological human tissue.

## 1. Introduction

Photoacoustic microscopy is an emerging biomedical imaging modality that has demonstrated success in visualizing cell nuclei, microvascular structures, and whole organs with rich contrast and high resolution [1,2]. Unlike fluorescence microscopy, photoacoustic microscopy does not require the use of exogenous dyes to provide contrast. Instead, it relies on the high endogenous optical absorption of biological tissue to form images. This enables the label-free recovery of biological chromophores. The contrast provided by optical absorption also exhibits high specificity which makes it possible to differentiate between chromophores [3]. In conventional optical-resolution photoacoustic microscopy (OR-PAM), a nanosecond pulsed excitation laser is tightly focused onto a target. As the target absorbs the pulses of light, the optical energy is converted to thermal energy. The energy conversion results in a rapid temperature change, generating ephemeral thermoelastic expansions. This creates ultrasound waves propagating in the sample which can then be detected at the surface of the sample using an ultrasonic transducer [4].

OR-PAM has demonstrated considerable success in achieving high-resolution imaging. The lateral resolution of an OR-PAM system is dependent on the excitation wavelength and the numerical-aperture (NA) of the optical objective. Transmission-mode OR-PAM has been efficacious in achieving submicron resolution by employing high NA objectives [5]. However, achieving submicron diffraction limited resolution in a reflection-mode configuration presents several challenges. For example, high NA objectives typically have small working distances, which makes it difficult to employ an acoustic-optical splitter to redirect the reflecting ultrasonic waves. While a thin piece of glass can be used as an acoustic-optical splitter, even low refractive index glass would exhibit some chromatic aberration, which greatly affects high NA objectives, ultimately reducing the effective resolution of the system [6]. Customized acoustic parabolic mirrors and ring transducers have been employed to provide enhanced resolution in reflection-mode OR-PAM [7,8]. However, such complex components increase the cost and complexity of the system.

A recently reported imaging modality known as photoacoustic remote sensing (PARS) replaces the ultrasonic transducer with a continuous-wave detection laser [9,10,11,12]. PARS, unlike most reflection mode OR-PAM systems, provides high-quality optical resolution photoacoustic imaging in a noncontact setting. In PARS imaging, the detection laser is cofocused with the excitation laser which enables optical interrogation of initial pressures within the excited region. The all-optical design of PARS does not require any additional components between the objective and the target, yielding a significantly simpler design. This also permits the use of high NA objectives to achieve high resolution in a label-free, noncontact reflection-mode configuration. As with the majority of optical-resolution photoacoustic microscopes, PARS relies on point by point excitation of chromophores to form images. A tightly focused beam would result in higher resolution but would also require a smaller raster scanning step size to achieve good sampling across the field of view [13].

Acquiring a large field of view with a small step size can be a time-consuming operation, dependent on scanning speed, laser repetition rate, and the acoustic and optical processes. However, high resolution large field of views are crucial for many applications in medical imaging. One important application is histopathological examination. Surgical oncologists excise diseased tissue for histopathological analysis of the surgical margins. To ensure negative surgical margins, the pathologist must examine several thin sections of resected tissue with sufficient resolution to distinguish cell nuclei. Previously, we reported a preliminary investigation into the visualization of cellular morphology in unstained human tissue and thick, formalin-fixed, embedded tissue blocks [14,15]. However, to visualize surgical margins in fresh tissue or in situ, it would be necessary to maintain subcellular resolution and recover cellular morphology in large field of views while operating in a reflection-mode configuration.

Previous works have utilized optical scanning to scan small regions rapidly [16]. In combination with a high repetition rate laser, a high point density can be achieved in a short amount of time. However, with this method, the field of view is often limited by the scanning speeds of the optomechanical systems. For example, galvanometer mirrors are typically only able to operate at a few hundred hertz with small swing angles. This limits the field of view that can be optically scanned on the sample to typically a few hundred microns, which may not be sufficient for clinical use. Mechanical stages enable the system to acquire significantly larger field of views, but move at a much slower pace due to mechanical limitations. Microlens arrays have also been utilized to significantly increase the effective area under imaging, but require careful management of heat deposition on the target [17].

Previously, works have reported mosaic acquisition methods for conventional photoacoustic systems [18,19]. Shao et al. reported a mosaic acquisition method for optical-resolution photoacoustic microscopy with a step size of 1.2 µm. The reported method is able to acquire a 6.45 × 5.8 mm^2^ field of view in ninety seconds [19]. However, as PARS has demonstrated submicron resolution in a reflection-mode configuration, it necessitates a method that is capable of rapidly acquiring large field of views while maintaining a small step size and high resolution [10]. The reflection-mode configuration also enables the imaging of thick targets, and is more analogous to in situ imaging. To take advantage of these capabilities, the method must allow for larger targets, such as animals or humans, to be scanned. Here, a method is described for fast and high-resolution mosaic acquisitions for PARS. This method enables the capture of large field of views with high point densities in a noncontact reflection-mode operation. A unique advantage of this method is that it minimizes the stage movement during scanning. This makes it possible to mount the optical components on top of the mechanical stages leading to the scanning mechanism, enabling wide field of view imaging of larger and heavier specimens. The method utilizes scanning mirrors to acquire small local frames and mechanical stages to move the sample in a cartesian grid pattern. This enables the capture of a sequence of images that are assembled together to form a larger field of view with a point density that is equal to an individual mirror scan. Images of carbon fiber networks and unstained histology samples are presented to demonstrate the method’s efficacy.

## 2. Materials and Methods

### 2.1. Experimental Apparatus

**Figure 1:**
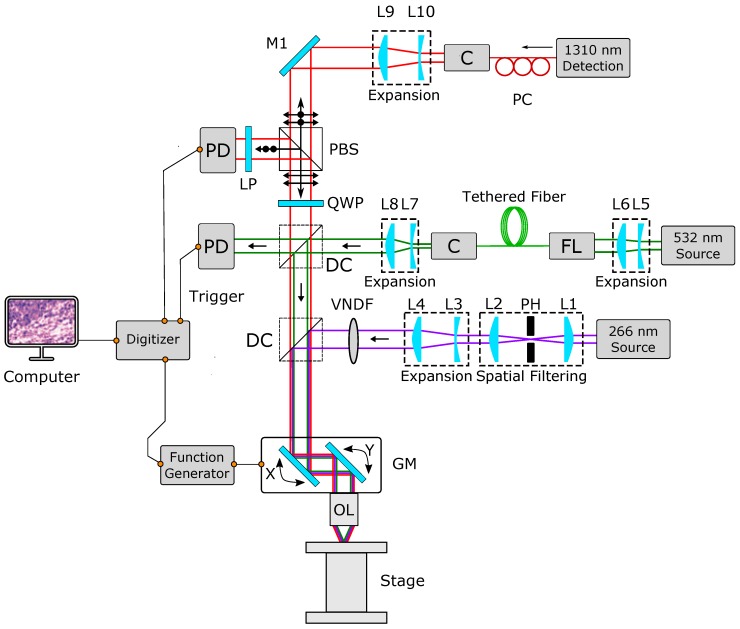
Schematic of the PARS microscope. Components labels are: pinhole (PH), variable neutral density filter (VNDF), collimator (C), polarized beam splitter (PBS), quarter waveplate (QWP), dichroic mirror (DC), photodiode (PD), fiber launch (FL), galvanometer mirrors (GM), lenses (L), mirrors (M).

The experimental setup is shown in Figure 1. This study uses two excitation sources; a 266 nm 0.5 ns pulsed laser with a 20 kHz repetition rate is used to image human histology samples, and a 532 nm 3 ns pulsed laser capable of 600 kHz repetition rate is used to demonstrate the system’s capability to capture fast mosaics. The 266 nm beam is spatially filtered using a 25 µm pinhole and collimated by two lenses. The collimated beam is expanded using a variable beam expander and combined with the 1310 nm and 532 nm beams using a dichroic mirror. The 532 nm beam is collimated and expanded to be coupled into an optical fiber. Then, a fiber-coupled collimator outputs the light which is further expanded to fill the aperture of the objective lens and combined with rest of the system with a dichroic mirror.

The detection beam is a 1310 nm continuous wave laser. The detection beam was vertically polarized using a fiber polarization controller. The polarized beam was collimated and expanded using a variable beam expander. The resultant beam was passed through a polarizing beam splitter and further passed through a quarter-wave plate in order to produce circularly polarized light. The coaligned detection and excitation beams were then directed to a set of 2D scanning mirrors (GVS412, Thorlabs Inc., Newton, NJ, USA). The scanning mirrors were mounted at right angles to each other and were able to steer the beam to any given point in the field of view. The amount and rate of swing is determined by the input waveform’s amplitude and frequency, respectively. The coscanned beam was targeted onto the tissue which was held on top of a set of *x-y* linear scanning stages (XM-100S, Newport Inc., USA). The back-reflected, circularly polarized light from the sample was sent back through the quarter-wave plate to be converted into vertically polarized light. The polarized beam splitter then reflected the vertically polarized light onto the photodiode (PDB425-C, Thorlabs Inc., Newton, NJ, USA). A four-channel data acquisition card (CSE161G4, DynamicSignals LLC, Lockport, IL, USA) was used to record the photoacoustic signal, along with the fast and slow mirror positions. The excitation source was used as the trigger for the data acquisition card which records the mirror signals for positional information and the PARS signals. The scanning mirrors were driven using a function generator and were not triggered or synchronized.

### 2.2. Mosaic Acquisition

Optical scanning forms the basis of mosaic acquisitions. A mosaic is formed by acquiring a sequence of frames constructed by optical scanning in a two-dimensional grid. A custom acquisition software was developed in C++ to interface with the data acquisition card and the mechanical stages. The software programs the data acquisition card with the desired frame point count, the length of time to record after each trigger, and the sampling rate. It also sets the speed of the mechanical stages and moves them in a two-dimensional grid to acquire mosaic frames. The software computes the grid’s coordinates based on the grid size, the field of view of each frame, and the desired overlap between each frame. The distance between each adjacent frame’s center depends on the required field of view per frame and the desired overlap. The degree of tilt of the scanning mirrors and the objective lens determine the field of view. The mirrors are controlled using two ramp waveforms fed from a function generator. In contrast to sinusoidal waveforms, the use of ramp waveforms yields a more consistent step size between each point acquisition. This is due to having a constant acceleration until the half-way point where the waveform flips and decreases again with a constant acceleration. In comparison, a sinusoidal waveform accelerates and decelerates at varying rates near the half-way point of the waveform. As a ramp waveform has a quick turnaround at the half-way point, it also results in less dwell time, limiting the heat dissipation on the sample. The amplitude and frequency of these mirrors, along with the laser repetition rate, determines the step size between each point acquisition. For example, with a repetition rate of 40 kHz, mirror frequencies of 120 mHz and 60 Hz yield a step size of 570 nm when the field of view is 380 × 380 µm^2^. Similarly, when the laser repeats at 600 kHz, mirror frequencies of 1.8 Hz and 960 Hz are used to yield a step size of 200 nm with a field of view of 121 × 121 µm^2^. The 960 Hz swing rate is under the bandwidth of the mirrors which are capable of a 1 kHz swing rate if mirror deflection is less than 0.2°.

Since PARS only relies on initial pressure measurements, the data acquisition card is programmed to record for a short amount of time after each trigger, i.e., typically only for 320 ns, resulting in significantly less data to be transferred [9,10]. Since the bandwidth of the photodiode is 75 MHz, a sampling rate of 200 MS/s suffices, resulting in 64 element segments for each point acquisition; this sampling rate was used for all acquisitions presented. The data acquisition card is utilized such that the raw measurements are streamed to the computer resulting in zero transfer time. Once the desired number of point acquisitions are acquired, the inhouse developed software writes the mirror signals and the time-domain PARS signals to disk. Once the data has been written, the software moves the mechanical stages such that the next frame in the sequence can be acquired, and enables the data acquisition card to begin the next scan. This movement is rapid, taking approximately 100 ms.

### 2.3. Mosaic Reconstruction

**Figure 2:**
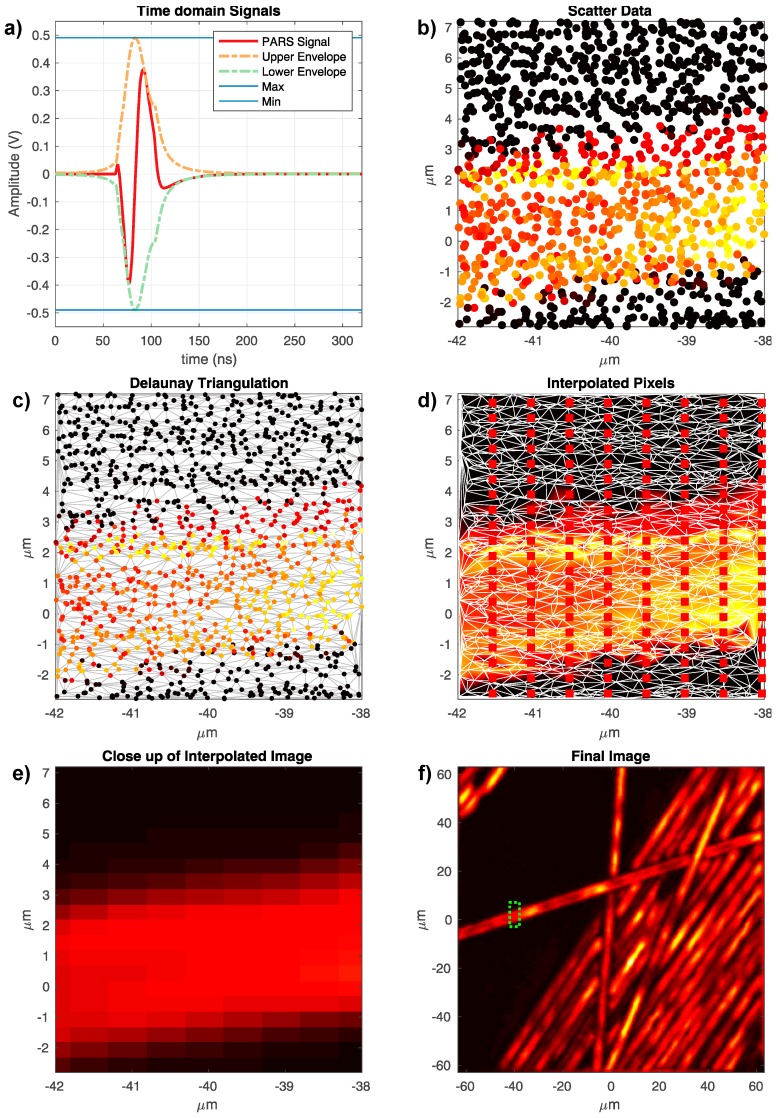
(**a**) PARS time-domain signal with upper and lower envelopes (**b**) raw scatter data (**c**) Delaunay triangulation (**d**) cartesian grid imposed on Delaunay triangulation (**e**) close up of interpolated pixels (**f**) reconstructed image.

Figure 2 provides a visual overview of the reconstruction process. The acquisition process results in a collection of points spread over the field of view. Each point has an *x* and *y* coordinate and a PARS signal associated with it (Figure 2a). The Hilbert Transform is employed to compute the upper and lower envelopes of the signal, shown as the orange and green dashed curves in Figure 2a [20]. The amplitude of these envelopes (blue lines) determines the intensity values of the scatter data (Figure 2b). To render an image, the scattered dataset is triangulated using Delaunay Triangulation (Figure 2c), which leads to optimized triangulation of the data and minimizes interpolation artifacts due to elongated triangles. This method has been widely used in the literature for surface reconstruction from scattered datasets [21,22]. Since the scatter data must be rendered on a cartesian grid to form an image, such a grid is imposed over the triangulation as an image, i.e., as points to be interpolated (Figure 2d).

Using linear interpolation, the intensity at each Cartesian point is then computed from the resulting triangulation. This results in a rendered image, as shown in Figure 2f. The Grid/Stitching plugin in Fiji software is used to assemble all the mosaics presented in this study [23]. This plug-in computes a correlation between the overlapping regions to calculate the best registration transformation. In addition, it applies a linear blend on the overlapping areas to smooth out any hard edges on adjacent frames.

### 2.4. Sample Preparation

Human breast tissue was obtained according to a protocol approved by the University of Waterloo Health Research Ethics Committee (Humans: #40275 Photoacoustic Remote Sensing (PARS) Microscopy of Surgical Resection, Needle Biopsy, and Pathology Specimens) and by the Research Ethics Board of Alberta (Protocol ID: HREBA.CC-18-0277). The specimen was obtained from fresh mastectomy resections, and was placed in formaldehyde to allow tissue fixation to occur. All experiments were done in accordance with the relevant guidelines and regulations. The samples were deidentified and no patient identifiers were provided to the researchers. As PARS allows for label-free imaging, an unstained slide was prepared by sectioning 4 µm thick tissue samples. These samples were placed on a glass slide and baked at 60 °C for an hour. An adjacent slide was prepared in the same fashion but was dyed with hematoxylin and eosin for comparison to the PARS image.

## 3. Results and Discussion

**Figure 3:**
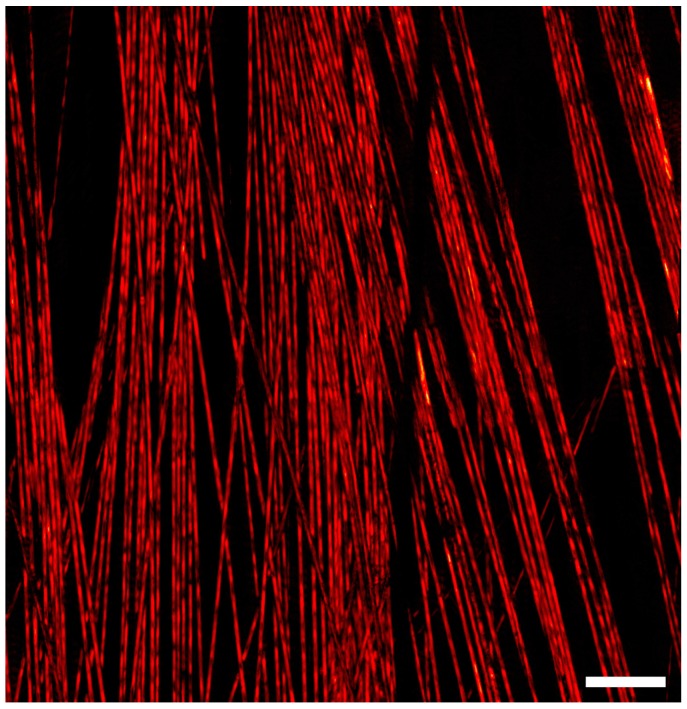
A 100-frame mosaic, arranged in a 10 × 10 grid. Each frame is 125 × 125 µm^2^, whereas the total field of view is approximately 0.8 × 0.8 mm^2^. The image was acquired with a 532 nm excitation source operating at 600 kHz. Scale bar 100 µm.

**Figure 4:**
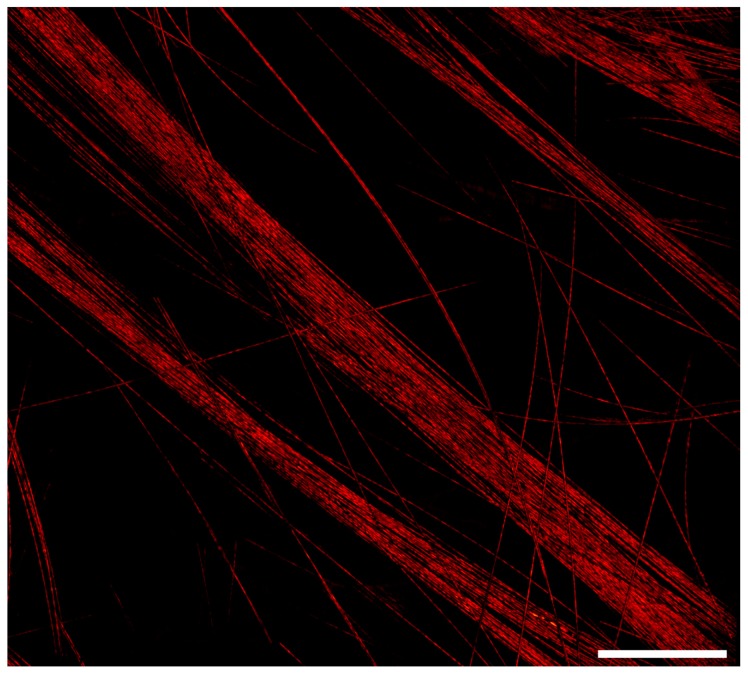
A 400-frame mosaic of carbon fiber networks, arranged in a 20 × 20 grid. Each frame has a field of view of 380 × 380 µm^2^, whereas the total field of view is 3 × 3 mm^2^. The image was acquired with a 532 nm excitation source operating at 40 kHz. Scale bar 500 µm.

To demonstrate this method, a 0.8 × 0.8 mm^2^ mosaic of carbon fiber networks composed of one hundred frames was acquired at a repetition rate of 600 kHz (Figure 3). Each frame covered an area of 121 × 121 µm^2^ and consisted of 400,000 raw point measurements. Operating at 600 kHz, the acquisition time for each frame was 0.66 s, with the total acquisition time amounting to seventy seconds. At such repetition rates, it was necessary for the mirrors to scan at 1.8 and 960 Hz. Operating at these frequencies poses two major limitations for acquisition in the current system. First, the scanning mirrors can only operate at low voltages, and hence, cover only a small field of view rapidly. Second, the high frequencies increase the heat generation of the control electronics significantly. A larger field of view can be obtained if sufficient active cooling is provided to the control electronics of the galvanometers. A wider field of view can be imaged in the same amount of time by employing a wide-angle lens or by reducing the number of raw measurements per frame. Figure 4 demonstrates the system’s ability to acquire a large field of view with a high number of frames. Four hundred frames were acquired with a 35% overlap to ensure good image registration. Each frame had a field of view of 380 × 380 µm^2^ and was composed of 400,000 raw data points. At 40 kHz, each frame took 10 s to acquire, with the total time being 66 min for the entirety of the mosaic.

**Figure 5:**
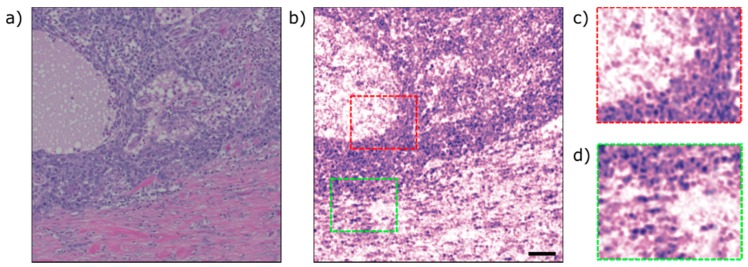
A comparison between a PARS image of unstained human breast tissue with conventional bright-field microscope. (**a**) a standard H&E stained image (**b**) a PARS mosaic of one hundred frames, total field of view of 1 × 1 mm^2^ (**c**,**d**) zoomed in versions of (**b**) showing cell nuclei. The image was acquired with a 266 nm excitation source operating at 20 kHz. Scale bar 100 µm.

To validate the method on tissue samples, we imaged human breast tissue with invasive ductal carcinoma (Figure 5)**.** Two tissue slides were prepared using adjacent sections from the formalin fixed paraffin block. One slide was stained with hematoxylin and eosin (H&E) dyes, and imaged using a conventional bright-field microscope (Figure 5a). The other slide was left unstained and imaged using the PARS microscope (Figure 5b). The PARS microscope utilizes a 266 nm laser to provide DNA constant. The PARS image was a mosaic of one hundred frames, with each frame being 85 × 85 µm^2^ and consisting of 100,000 points. The total field of view was approximately 1 × 1 mm^2^ and took 470 s to acquire. The acquisition time in this case was limited by the 21 kHz repetition rate of the 266 nm laser. Figure 5c,d show zoomed-in versions of the assembled mosaic visualizing cell nuclei of cancerous and healthy human cells. The results of the proposed system are summarized in Table 1.

Previously, Hu et al. have reported an acquisition time of 70 min for an area of 7.8 × 10 mm^2^ with mechanical stages [24]. However, the reported acquisition utilized a step size of only 2.5 µm. With a similar step size, the proposed method in this work would acquire a single frame with a field of view of 380 × 380 µm^2^ in ~0.5 s. To acquire a 10 × 10 mm^2^ image, the total acquisition time would be 7 min at a 40 kHz repetition rate. With a 600 kHz repetition rate, the total acquisition time would reduce to approximately 26 s, yielding a significantly faster system which is vital for point by point scanning microscopes. The significant increase in speed seen in this work can be traded for a smaller step size resulting in higher sampling resolution. Similarly, other works on the subject have reported results with a limited repetition rate, step size and resolution as compared to this work [19]. Employing a wide-angle lens, each frame is acquired in one second and covers a field of view of 930 × 930 µm^2^ consisting of 160,000 measurements. The increased spot size allows for a step size of 1.2 µm between point acquisitions lowering the number of points required per frame at the cost of resolution. In contrast, here we are able to acquire a 400,000 point frame in 700 ms, including the acquisition and stage movement time at a resolution of 1.2 µm and a step size of 200 nm. The 600 kHz repetition rate halves the acquisition time while the transfer time is eliminated completely by streaming the data while the scan is in progress. Employing a similar wide-angle objective that results in a frame size of 930 × 930 µm^2^, an area of 9 × 9 mm^2^ can be acquired in seventy seconds with the same point count per frame. A lower point count will further increase the rate of the acquisition.

## 4. Conclusions

This study demonstrated a method for PARS to enable large field of view acquisitions with a high resolution and small step size to be acquired rapidly. Carbon fiber networks were presented with large field of views and high repetition rates. The method was further demonstrated on human breast tissue, which showed good agreement with the cellular morphology in the H&E samples. In conclusion, the presented method has several advantages. The speed enhancement makes it possible to acquire images twice as quickly compared to previous methods, while the small step size combined with high spatial resolution yield high quality images. Moreover, by minimizing the stage movement, it allows for delicate optics to be mounted on the stages, making it possible to image heavier targets. The authors believe that the rapid acquisition of large field of views combined with high resolution and a small step size can aid clinicians and makes human in situ imaging practical.

## Figures and Tables

**Table 1:** Summary of results of the proposed system.

	Field of View	Step Size	Point Count	Rep. Rate	Acquisition Time
**Proposed Method Figure 3**	0.8 × 0.8 mm^2^	200 nm	400,000	600 kHz	70 s
**Proposed Method Figure 4**	3.0 × 3.0 mm^2^	600 nm	400,000	40 kHz	66 min
**Proposed Method Figure 5**	1.0 × 1.0 mm^2^	260 nm	100,000	21 kHz	8 min

## References

[1] Yao D.-K., Chen R., Maslov K., Zhou Q., Wang L.V. (2012). Optimal ultraviolet wavelength for in vivo photoacoustic imaging of cell nuclei. J. Biomed. Opt..

[2] Wong T.T.W., Zhang R., Zhang C., Hsu H.-C., Maslov K.I., Wang L., Shi J., Chen R., Shung K.K., Zhou Q. (2017). Label-free automated three-dimensional imaging of whole organs by microtomy-assisted photoacoustic microscopy. Nat. Commun..

[3] Beard P. (2011). Biomedical photoacoustic imaging. Interface Focus.

[4] Yao J., Wang L.V. (2013). Photoacoustic Microscopy. Laser Photon Rev..

[5] Zhang C., Maslov K., Wang L.V. (2010). Subwavelength-resolution label-free photoacoustic microscopy of optical absorption in vivo. Opt. Lett..

[6] Rao B., Li L., Maslov K., Wang L. (2010). Hybrid-scanning optical-resolution photoacoustic microscopy for in vivo vasculature imaging. Opt. Lett..

[7] Zhang C., Maslov K., Hu S., Chen R., Zhou Q., Shung K.K., Wang L.V. (2012). Reflection-mode submicron-resolution in vivo photoacoustic microscopy. J. Biomed. Opt..

[8] Yao D.-K., Maslov K., Shung K.K., Zhou Q., Wang L.V. (2010). In vivo label-free photoacoustic microscopy of cell nuclei by excitation of DNA and RNA. Opt. Lett..

[9] Hajireza P., Shi W., Bell K., Paproski R.J., Zemp R.J. (2017). Non-interferometric photoacoustic remote sensing microscopy. Light Sci. Appl..

[10] Reza P.H., Bell K., Shi W., Shapiro J., Zemp R.J. (2018). Deep non-contact photoacoustic initial pressure imaging. Optica.

[11] Bell K.L., Hajireza P., Shi W., Zemp R.J. (2017). Temporal evolution of low-coherence reflectrometry signals in photoacoustic remote sensing microscopy. Appl. Opt. AO.

[12] Bell K., Hajireza P., Zemp R. (2018). Scattering cross-sectional modulation in photoacoustic remote sensing microscopy. Opt. Lett. OL.

[13] Wang L.V. (2017). Photoacoustic Imaging and Spectroscopy.

[14] Abbasi S., Le M., Sonier B., Dinakaran D., Bigras G., Bell K., Mackey J.R., Reza P.H. (2019). All-optical Reflection-mode Microscopic Histology of Unstained Human Tissues. Sci. Rep..

[15] Abbasi S., Abbasi S., Le M., Le M., Sonier B., Bell K., Bell K., Dinakaran D., Dinakaran D., Bigras G. (2019). Chromophore selective multi-wavelength photoacoustic remote sensing of unstained human tissues. Biomed. Opt. Express BOE.

[16] Xie Z., Jiao S., Zhang H.F., Puliafito C.A. (2009). Laser-scanning optical-resolution photoacoustic microscopy. Opt. Lett. OL.

[17] Jeon S., Kim J., Lee D., Baik J.W., Kim C. (2019). Review on practical photoacoustic microscopy. Photoacoustics.

[18] Baik J.W., Kim J.Y., Cho S., Choi S., Kim J., Kim C. (2019). Super Wide-field Photoacoustic Microscopy of Animals and Humans In Vivo. IEEE Trans. Med. Imaging.

[19] Shao P., Shi W., Chee R.K., Zemp R.J. (2012). Mosaic acquisition and processing for optical-resolution photoacoustic microscopy. J. Biomed. Opt..

[20] Jiang H. (2018). Photoacoustic Tomography.

[21] Amidror I. Scattered Data Interpolation Methods for Electronic Imaging Systems: A Survey. https://infoscience.epfl.ch/record/99883.

[22] Van Kreveld M., Schwarzkopf O., de Berg M., Overmars M., de Berg M. (2000). Computational Geometry: Algorithms and Applications.

[23] Preibisch S., Saalfeld S., Tomancak P. (2009). Globally optimal stitching of tiled 3D microscopic image acquisitions. Bioinformatics.

[24] Hu S., Maslov K., Wang L.V. (2011). Second-generation optical-resolution photoacoustic microscopy with improved sensitivity and speed. Opt. Lett..

